# Targeting Adenosine Signalling in Knee Chondropathy: The Combined Action of Polydeoxyribonucleotide and Pulsed Electromagnetic Fields: A Current Concept Review

**DOI:** 10.3390/ijms241210090

**Published:** 2023-06-13

**Authors:** Lorenzo Moretti, Davide Bizzoca, Alessandro Geronimo, Andrea Michele Abbaticchio, Francesco Luca Moretti, Arianna Carlet, Francesco Fischetti, Biagio Moretti

**Affiliations:** 1 Orthopaedics Unit—UOSD Vertebral Surgery, AOU Consorziale Policlinico, Piazza Giulio Cesare 11, 70124 Bari, Italy; lorenzo.moretti@libero.it; 2 Ph.D. Course in Public Health, Clinical Medicine and Oncology, University of Bari “Aldo Moro”, Piazza Giulio Cesare 11, 70124 Bari, Italy; 3 Orthopaedics Unit, DiBraiN, University of Bari “Aldo Moro”, Piazza Giulio Cesare 11, 70124 Bari, Italy; 4 National Centre for Chemicals, Cosmetic Products and Consumer Protection, National Institute of Health, 00161 Rome, Italy; 5 Departement DiBraiN, University of Bari “Aldo Moro”, Piazza Giulio Cesare 11, 70124 Bari, Italy

**Keywords:** knee chondropathy, polydeoxyribonucleotide (PDRN), Pulsed Electromagnetic Fields, PEMF, A2A receptor, adenosine signalling

## Abstract

Chondropathy of the knee is one of the most frequent degenerative cartilage pathologies with advancing age. Scientific research has, in recent years, advanced new therapies that target adenosine A2 receptors, which play a significant role in human health against many disease states by activating different protective effects against cell sufferance and damage. Among these, it has been observed that intra-articular injections of polydeoxyribonucleotides (PDRN) and Pulsed Electromagnetic Fields (PEMF) can stimulate the adenosine signal, with significant regenerative and healing effects. This review aims to depict the role and therapeutic modulation of A2A receptors in knee chondropathy. Sixty articles aimed at providing data for our study were included in this review. The present paper highlights how intra-articular injections of PDRN create beneficial effects by reducing pain and improving functional clinical scores, thanks to their anti-inflammatory action and the important healing and regenerating power of the stimulation of cell growth, production of collagen, and the extracellular matrix. PEMF therapy is a valid option in the conservative treatment of different articular pathologies, including early OA, patellofemoral pain syndrome, spontaneous osteonecrosis of the knee (SONK), and in athletes. PEMF could also be used as a supporting therapy after an arthroscopic knee procedure total knee arthroplasty to reduce the post-operative inflammatory state. The proposal of new therapeutic approaches capable of targeting the adenosine signal, such as the intra-articular injection of PDRN and the use of PEMF, has shown excellent beneficial results compared to conventional treatments. These are presented as an extra weapon in the fight against knee chondropathy.

## 1. Introduction

Knee osteoarthritis (OA) and chondropathy are the most frequent degenerative cartilage pathologies in elderly people [1].

Osteoarthritis (OA) is the most important and frequent joint pathology on a degenerative basis, which leads to pain and disability in elderly people. OA has been defined by The Osteoarthritis Research Society International (OARSI) as “a disorder involving movable joints characterized by cell stress and extracellular matrix degradation initiated by micro-and macro-injury that activates maladaptive repair responses including pro-inflammatory pathways of innate immunity” [1].

OA represents an important social health problem, affecting people worldwide, but it is mainly prevalent among adults over 65 years [2].

Osteoarthritis is responsible for articular pain, joint stiffness, and loss of function, thus progressively affecting the patient’s working ability and social life [3,4,5]. Nonetheless, the onset of the symptoms occurs after the irreversible joint changes have developed [2]. Therefore, arthroplasty is still the most successful procedure for the treatment of OA, with a reported high patient satisfaction rate [1,2].

Chondropathy consists of the suffering of the articular cartilage, which could appear thinned and/or have deep fissures that could reach the subchondral bone; it is the antechamber of osteoarthritis in many patients [1,2]. Chondropathy of the knee causes intense pain, joint stiffness, and loss of functionality, thus affecting the quality of life of the patient who suffers from it, limiting their working activity and social life. Chondral lesions can lead to an excessive load on the subchondral bone and, thus, to bone edema, which can manifest itself with painful symptoms and limit physical activity [1,2].

Over time, bone edema can resolve if adequately treated or evolve towards spontaneous osteonecrosis of the knee (SONK), which can also be secondary to vascular pathologies, or towards arthritic evolution [1,2].

The classic conservative therapeutic approaches to combat this pathology consist of analgesics, non-steroidal anti-inflammatory drugs (NSAIDs), and intra-articular injections of corticosteroids and hyaluronic acid (HA) [1,2].

Although the effectiveness of the therapeutic approaches has been abundantly demonstrated, the most recent scientific studies have brought to light alternative methods to target knee chondropathies, such as injections of PDRNs and PEMF [3].

Adenosine A2 receptors (A2A), members of the G protein-coupled receptor family, contribute to increasing coronary circulation by vasodilation and immune cell suppression, thereby protecting tissue from inflammation [3].

A2A receptors are expressed in several cell lines, such as chondrocytes and synoviocytes, with variable density. In particular, A2A and A3Ars are increased in the presence of PEMF. 

This review aims the depict the role and therapeutic modulation of A2A receptors in knee chondropathy.

## 2. Methods

The first step consisted of a scoping literature search performed on studies published from January 2002 to August 2022 by two reviewers, A.A. and A.G., using the following databases: OVID-MEDLINE^®^, EMBASE, SCOPUS, Web of Science, Google Scholar, and PubMed. We selected an initial pool of potentially relevant studies, aiming at studying polydeoxyribonucleotide and Pulsed Electromagnetic Fields as possible therapies in the context of knee chondropathy and osteoarthritis, targeting the adenosine A2 receptors.

The search strategy included the following terms: “adenosine signalling, A2A receptors, knee chondropathy, polydeoxyribonucleotide, Pulsed Electromagnetic Fields”.

Inclusion criteria were clinical trials (using the query “Clinical Trial” in the search engine) and systematic reviews focusing on the treatment of knee chondropathy. These were sought, in particular, by entering the terms "PDRN", "PEMF" and "A2A receptors" in the aforementioned scientific search engines; furthermore, we selected recent articles (from 2002 onwards) to give greater relevance to our scientific purpose. The exclusion criteria were: articles published before 2002 and articles that did not mention the A2A receptor pathways or the types of knee OA treatments with PDRN, PEMF, or innovative alternative therapies other than the traditional ones.

The database search provided a total of 98 studies for potential inclusion in the review. After adjusting for duplicates, 57 studies remained. Of these, 3 studies were discarded after reading titles and reviewing abstracts. A total of 6 additional abstracts were identified by checking the references of the relevant papers. A total of 60 articles were finally included in the present review.

## 3. Results

### 3.1. A2A Receptors 

Adenosine plays a significant role in defending human health against many pathologies by exerting several protective effects against cell damage, in both the brain and the periphery, through interaction with A1, A2A, A2B, and A3 adenosine receptors (ARs) [4].

In clinical biophysics, low-frequency low-energy pulsed electromagnetic fields (PEMF) are a fundamental tool for investigating the development and the importance of physical stimuli in the control of biological activities. Many in vivo or in vitro studies have been performed to identify the biophysical stimulation induced by PEMF as a potential alternative to pharmacological treatments in several inflammatory-related pathologies [5,6,7].

Cartilage lesions are reported to represent a major health problem and contribute to the highest rate of world disability due to the body’s limited cartilage regeneration capability. In recent decades, several studies have been developed to resolve this cause of disability, some of them with physical stimuli approaches. From a cellular point of view, a wide body of in vitro studies has investigated the effect of PEMF on chondrocytes and synoviocytes.

ARs are expressed in these cell lines with variable density. In the presence of PEMFs, only A2A and A3ARs are increased, whereas A1 and A2BARs show binding parameters similar to those of control cells [8]. After PEMF treatment, A2A and A3AR agonists reveal an amplified effect on cAMP production that is blocked by selective A2A and A3AR antagonists [8].

The PEMF’s transmembrane signal recognition processes were first reported by Varani et al. [9]. These authors found that ARs were the primary target of PEMF stimulation in inflammatory cells; ARs play a major role in the control of inflammatory processes, with both pro-inflammatory and anti-inflammatory effects. PEMF exposure has been shown to increase A2A and A3AR density in cell membranes of osteoblasts, chondrocytes, and synoviocytes [10]. In addition, it inhibits IL-6 and IL-8 cytokines, while stimulating the release of the anti-inflammatory cytokine IL-10 and inhibited prostaglandin E2 (PGE2) production with up-regulation of A (2A) receptors [11]. IL-1β is a pro-inflammatory cytokine that promotes ECM cartilage degradation in healthy and osteoarthritic-joint-derived cells. In a study performed on cartilage explants, it was reported that PEMF inhibits the negative effect of IL-1β cytokine [12].

### 3.2. Polydeoxiriboneucleotides

**Figure 1:**
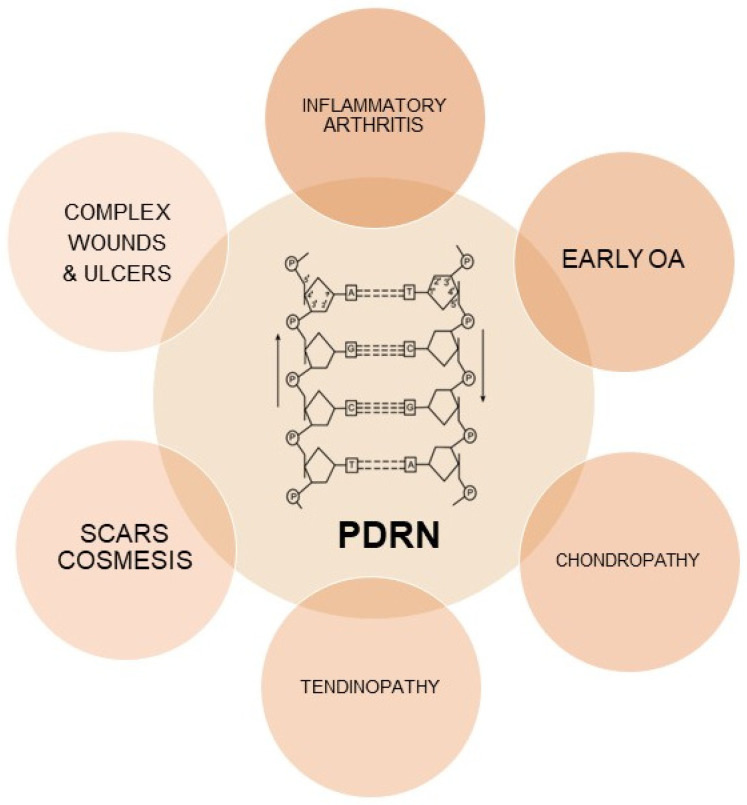
PDRN structure and therapeutic implications.

Polydeoxyribonucleotides (PDRNs) are a family of DNA-derived drugs, characterized by a molecular weight of 50–1500 kDa, that derive from a standardized process of purification and sterilization of sperm DNA from Oncorhynchus mykiss (Salmon Trout) or Oncorhynchus keta (Chum Salmon) (Figure 1) [13].

They have been shown to have a crucial role in suppressing the release of pro-inflammatory cytokines and activating anti-inflammatory effects. Moreover, they have been proven to have various other beneficial effects, such as stimulating wound healing, tissue repair, and anti-ischemic action [14].

PDRN is an agonist of one of the adenosine-activated receptors, the A2A receptor. Adenosine binds and activates four specific adenosine receptors referred to as A1, A2A, A2B, and A3.

The A2A receptor plays a crucial role in the regulation of inflammation, cell growth, angiogenesis, oxygen consumption, and cellular ischemia. 

As demonstrated by Thellung et al., PDRN induces cell growth in the primary cultures of human skin fibroblasts [13].

As highlighted, the incubation with 3,7-dimethyl-1-propargyl xanthin (DMPX), which is an adenosine A2 receptor antagonist, inhibited the action of PDRN, thus suggesting that PDRN may act preferentially on the A2A receptors [14].

Normally, damaged or ischemic tissue cannot be subjected to “de novo” DNA synthesis. Instead, as highlighted by Sini et al. [15], PDRN generates nucleosides and nucleotides that may be involved in DNA formation, thus stimulating the reactivation of physiological cell proliferation and its regular growth patterns.

In addition to fibroblasts, the agonist effect of PDRN on cell growth has been reported in cultures of osteoblasts and chondrocytes [16,17]. In the latter, it has been seen that it induces a physiological accumulation of extracellular matrix and an inhibition in the activity of matrix metalloproteinases, thus, leading to reduced degradation of proteoglycans [18].

At the genome level, it has been shown that PDRN has an inhibitory action on gene expression in the extracellular matrix, leading to a low rate of its degradation.

This scientific evidence has laid the foundations for the development of DNA-derived drugs as an emerging therapy in the regenerative therapy of suffering and damaged cartilage.

Additionally, PDRN has been shown to speed up bone tissue regrowth and repair [16].

The upregulation of CD31 expression, angiopoietin, and transglutaminase-II, factors that are involved in the formation of new vessels, demonstrated that PDRN is also implicated in increased angiogenesis.

Furthermore, PDRN reduces blood levels of tumour necrosis factor-α (TNF-α) and increases the release of VEGF and nitric oxide (NO) in wounds, showing a significant systemic effect [19]. 

As demonstrated by Bitto et al., PDRN can promote angiogenesis in an in vitro model of peripheral artery occlusive disease [19].

Activation of the adenosine A2A receptor has been shown to have anti-inflammatory potency and stands as an attractive target for anti-inflammatory markers.

Recent literature shows how PDRN improves the clinical signs of arthritis, decreases histological damage by restoring histological features, reduces the amount of many pro-inflammatory and apoptotic cytokines both in the blood and in the cartilage itself, and, on the other hand, increases the expression of anti-inflammatory cytokines such as interleukin-10 (IL-10) [20].

As regards the practical purposes of its use, the A2A receptor has been designated as a therapeutic target to modulate inflammatory and ischemic damage. Its use has been suggested through studies conducted on experimental models in the urological field for the treatment of testicular torsion and varicocele [21,22], where it has been demonstrated that PDRN may decrease the inflammatory pathway and equilibrate the pro-inflammatory and apoptotic process, thus stimulating spermatogenesis and protecting the tissues from the histological sufferance and lesions. The latter aspect may also be referred to as the capacity of PDRN to significantly reduce ischemic reperfusion damage by increasing VEGF expression and therefore angiogenesis [23].

Initially, PDRN was introduced in the dermatological field for the treatment of skin pathologies such as skin graft donor sites, photorefractive keratectomy, and radio dermatitis [24].

Regarding safety, in in vivo studies PDRN does not increase the risk of mortality or have any toxic effects on the lungs, liver, heart, brain, or skeletal muscles [24,25].

In clinical trials, PDRN has shown excellent safety and tolerability [26,27,28,29,30,31,32,33,34,35,36,37,38,39,40,41,42,43,44,45]. Therefore, a post-marketing surveillance study involving selling more than 300,000 PDRN-dispensed prescriptions confirmed the significant safety profile of the drug [45]. In the literature, there are no data and reports of side effects associated with the use of intra-articular injections of PDRN. However, due to the absence of scientific data to support its use during pregnancy or while breastfeeding, its use is not recommended. Furthermore, its use is contraindicated in subjects allergic to PDRN, although there are no allergies reported in the literature to this drug.

#### PDRN in Chondropathy

As has been shown, polynucleotides are polymeric molecules with a viscoelastic property because they bind large quantities of water molecules [16,25,26,27]. This allows them, as part of the treatment of osteoarthrosis (OA), to have the capacity to reorganize the articular cartilaginous structure through the regulation of the coordination and orientation of water molecules, thus increasing the moisture of the articular surfaces when the PDRN is injected into the joints [17].

As mentioned above, PDRN can inhibit metalloproteases of the extracellular matrix, thus reducing the degradation of extracellular matrix proteoglycans.

Polynucleotides are also correlated to stimulating cell growth, collagen production, migration of anti-inflammatory cell types, and decreasing inflammation.

As highlighted by Bitto et al., PDRN injections have been shown to significantly improve OA symptoms, reduce the expression of pro-inflammatory factors such as tumour necrosis factor-alpha (TNF-alpha) and interleukin 6 (IL-6), and, on the other hand, increase the expression of anti-inflammatory factors such as interleukin 10 (IL-10) [20]. The main biological effects of PDRN are mediated by the interaction with A2A receptors [20].

There have been several studies in the literature which have compared the therapeutic effects of the intra-articular injection of PDRN with hyaluronic acid (HA) injections in patients suffering from knee OA, and Kim et al. collected them in their Systematic Review and Meta-Analysis [28]. What we have seen is that in the first 2 months after the infiltration, patients subjected to PDRN intra-articular injection showed less pain, in terms expressed according to the VAS score (Visual Analogue Scale), than those treated with HA. From 4 months post-treatment onwards, no significant difference was seen between the PDRN and HA groups [27,29,30].

As far as functionality is concerned, the scores that were used for its evaluation were the KOOS and the KSS. It was possible to appreciate notable improvements both in patients treated with HA and in those treated with PDRN without, however, observing significant differences between the two groups.

However, it was demonstrated that the injection of PDRN led to a better pain relief effect, superior to HA for 2 months post-injection [29].

Consequently, Dallari et al. proposed to combine, in the same formulation of the preparation to be injected into the joint, both HA and PDRN (PNHA) [29].

This combination stimulates greater ECM production and synoviocyte growth than injections of HA or PDRN alone. In this way, what is obtained is a combination of the trophic effect of PDRN and the viscoelastic action of HA, which turns out to be a useful feature in intra-articular treatments.

As it has been possible to deduce from the recent scientific literature, there is always a more significant interest in the study of biomarkers in the synovial fluid (SF). Research in the field of proteomics has set itself the goal of studying how, in OA, there are alterations in the expression of some specific SF markers [31]. Few studies have dosed these biomarkers at time 0 and subsequently in the various controls foreseen by the follow-ups. This is probably due to the scarcity of test tubes and specific reagents to collect the samples and to the greater "invasiveness" of the exam compared to oral questionnaires. However, from what could be deduced, in patients treated with PNHA there was a significant reduction in type 1 and type 3 metalloproteinases (MMP), proinflammatory cytokine interleukin 6 (IL-6), tumour necrosis factor-a (TNF-a), and prostaglandin E2 (PGE2). In patients treated only with HA, a reduction of the expression of IL-6, IL-8, and PGE2 only was observed [29].

### 3.3. PEMF

Biophysical stimulation is a non-invasive therapeutical approach increasingly used in orthopaedic clinical practice to improve the reparative capacities of the musculoskeletal system. It consists of the application of physical energy to a biological system to increase and facilitate tissue regeneration and anabolic activity [31].

Pulsed Electromagnetic Fields (PEMF) are low-frequency magnetic fields, with specific waveforms and amplitude that have constant variations of the magnetic field amplitude over time [46].

Different types of non-invasive electrical stimulation devices received approval from US FDA for clinical use in orthopaedics and traumatology, such as ultrasound energy (low-intensity pulsed ultrasound system, LIPUS), electrical energy applied directly to the tissue by adhesive electrodes (capacitively coupled electric fields, CCEF), and electromagnetic energy applied by coils (Pulsed Electromagnetic Fields, PEMF) [32].

**Figure 2:**
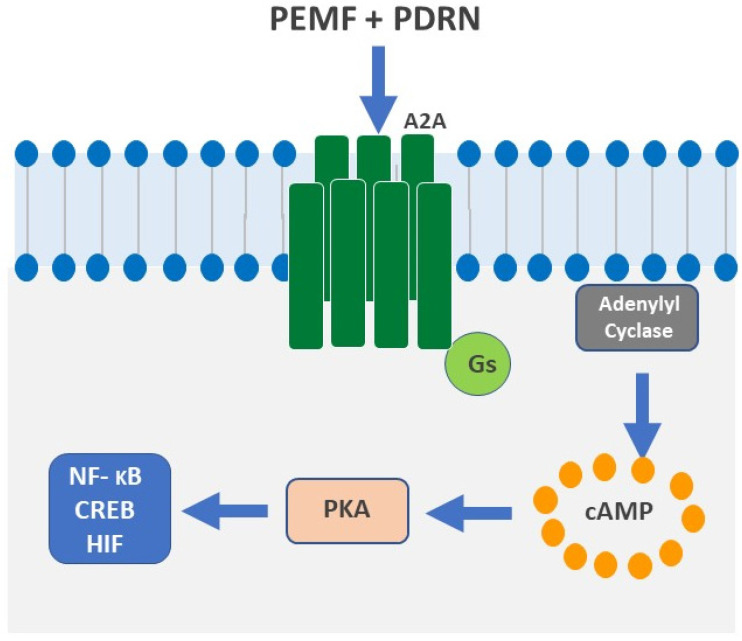
PEMF and PDRN synergism on the A2A receptors’ intracellular signalling. The figure shows the synergic activation of A2A receptors by PEMF and PDRN. A2A receptors belong to the G protein-coupled receptor (GPCR) family and are coupled with G stimulatory proteins (Gs). PEMF and PDRN activate the A2A receptor, thus causing Adenyl cyclase activation. The subsequent cyclic Adenosine Monophosphate (cAMP) intra-articular concentration finally leads to Protein Kinase A (PKA) activation, which causes, in turn, specific intracellular activation pathways (NF-KB: nuclear factor kappa-light-chain-enhancer of activated B cells; CREB: cAMP response element-binding protein; HIF: Hypoxia-inducible factor).

An electromagnetic field is a physical phenomenon resulting from the combination of an electric field and a magnetic field. The PEMF device produces a magnetic field that, in turn, induces currents, which flow into the nearby tissues [33]. These induced currents mimic the natural electrical activities that are physiologically induced within bones during movements, thus triggering a cascade of pathways from the cell membrane to the nucleus and at the gene level, where specific changes occur (Figure 2) [34,35].

PEMF’s mechanism of action, studied in the MC3T3-E1 cell line, involves the ryanodine-sensitive calcium channels’ activation in the sarcoplasmic reticulum and human chondrocytes. They also act by activating and upregulating mainly A2A and A3 adenosine receptor subtypes, thus leading to an anti-inflammatory effect due to the activation of the NF-kB pathway [7]. This interesting biological effect could explain the efficacy of PEMF in reducing articular inflammation.

Several pre-clinical and clinical trials have shown that PEMFs have positive effects on joint cartilage, synovia, and subchondral bone. In vitro and in vivo studies on animal cartilage cells, such as bovine, equine, or guinea pig cells [6,7,8], have been followed by subsequent studies performed on human mesenchymal cells (MSCs): it was found that chondrogenic differentiation of MSCs is facilitated upon PEMF exposure of varying amplitude and intensity [36]. 

Biophysical PEMF stimulation increases the chondrocytes’ proliferation in patients without OA [37] and upregulates the expression of growth factors, cytokines, and ECM components, such as glycosaminoglycans (GAGs), proteoglycans (PGs), and collagen II (COLLII) [38,39,40,41].

Moreover, some studies have investigated how PEMF influence the regeneration of chondrocytes isolated from subjects with OA. Stolfa and collaborators conducted experiments with different PEMF signal features and chondrocyte concentrations. These authors showed that PEMF have a positive effect on chondrocyte metabolism, but there were no significant effects on cell proliferation. However, these results were not repeatable in all experiments [19]. Hence, Schmidt-Rohlfing et al. reported that PEMF and sinusoidal magnetic fields have no effects on the metabolism of human osteoarthritic chondrocytes cultured in vitro in a collagen gel [42].

PEMF’s therapeutical frequency is also a matter of debate; in subjects with advanced phases of knee osteoarthritis, PEMF frequencies between 37 and 75 Hz were revealed to be effective in preserving the structural parameters of both cartilage and bone. However, stimulation at 75 Hz significantly improved the chondroprotective effect when compared to 37 Hz [43].

The combined effects of bone marrow concentrate (BMC) and PEMF in the healing of osteochondral defects, treated with a scaffold, have been evaluated in animal models in different studies. Both cellular and cartilage matrix parameters improved with the addition of PEMF stimulation compared to using the scaffold alone; the combination with BMC also facilitated osteochondral regeneration [44].

Biophysical therapy with specific and tested parameters of PEMF must be considered a valid aid to arthroscopic surgical treatment because of the role of cell stimulation and the reduction of inflammation and pain after treatment. Its use would allow an athlete a more rapid functional recovery and, therefore, the possibility of quickly coming back to sporting activity. However, unlike bone edematous pathology, in which it occupies a prominent place in association or not with bisphosphonates and load reduction, no studies are reported in the literature on sportsmen that evaluate whether biophysical therapy alone can replace surgical treatment in the case of mild/moderate chondral damage [47]. 

In the literature, no data are reported, to date, on side effects associated with excessive exposure to the PEMFs. However, certain precautions should be taken in particular conditions before proceeding with intra-articular PEMF therapy. The next paragraph will depict these conditions. 

### 3.4. PEMF in Chondropathy

Based on these preclinical findings, in clinical practice, PEMF could be successfully used as (i) post-surgical treatment, to quickly control local joint inflammation and, over the long term, to maintain the mechanical and biological properties of the cartilage or engineered tissue. These tissues could be used after arthroplasty to reduce inflammatory processes involving particular tissues and attenuate the possibility of developing chronic pain or functional limitations [45,48]; (ii) a conservative treatment to limit the progression of bone oedema, or in association with surgery for risk fractures, delayed union, and non-union.

Articular cartilage damage is strongly identified as a source of joint limitation and reduced performance in athletes, whether isolated or in conjunction with ligament or meniscal or tendon tears [49]. Therefore, surgical treatment should be supported by biophysical therapy to support functional recovery and achieve better outcomes.

The positive effects of PEMF, associated with some conservative therapies, on the quality of life in patients with knee osteoarthritis were evaluated in a meta-analysis [50]. Later, other groups evaluated their use of PEMF in the treatment of early osteoarthritis (Kellgren Lawrence < 2) at age < 60 for 2 years. The results were mixed, as they showed an improvement in pain symptoms, KOOS, and Tegner scores after one year of treatment and a worsening, instead, at two years [51]. The authors concluded that an annual repetition of the treatment may result in sustained symptomatic improvement for the patient. In another prospective level IV study, the same authors enrolled 22 patients with a mean age of 48.4 years and with early OA. They found that at the 1-year follow-up, a statistical improvement of KOOS, EQ-5D, Tegner score, and IKDC after PEMF treatment for 45 days [52]. Significant results were also highlighted by Iammarrone et al. from young patients with patellofemoral pain syndrome (PFPS) [53]. 

Marchegiani Muccioli et al., in a study based on 28 patients with spontaneous osteonecrosis of the knee (SONK), evaluated the clinical and MRI effectiveness of PEMF therapy used 6 h daily for 90 days. At the 6-month follow-up, a clinical improvement and a reduction of the SONK area were reported by MRI [54]. 

Collarile et al., in a prospective comparative study recruiting thirty patients suffering from grade III and IV International Cartilage Repair Society chondral lesions of the Knee treated with matrix-assisted autologous chondrocyte implantation (MACI), showed that the patients who received postoperative PEMF therapy reported a better clinical outcome both in the short- and long-term follow-up [34]. These findings reveal that biophysical stimulation is an effective approach that can improve regenerative medicine’s clinical results [55]. 

Cadossi et al. reported similar findings in another study based on 30 patients with grade III and IV Outerbridge osteochondral lesions of the talus (OLT) treated with a collagen scaffold seeded with bone-marrow-derived cells (BMDCs) [56]. These authors reported that the patients who randomly received postoperative PEMF stimulation revealed a better clinical outcome, assessed using the American Orthopaedic Foot and Ankle Society (AOFAS) score, Visual Analog Scale (VAS), and Short Form-36 (SF-36), at 12 months after surgery [56]. The authors concluded that PEMFs are useful in speeding up the patient’s recovery after BMDC transplantation [56]. 

Benazzo et al. reported reduced use of NSAIDs and more rapid recovery compared to the control group in patients who, after a cruciate reconstruction, had been managed with a pulsed magnetic field; however, there was no statistical improvement in IKDC and SF-36 [56]. 

Gremion et al. and Ozgüçlü and colleagues found that a different pulsed signal therapy improved the clinical state of treated patients, but there was no significant statistical difference between this and other conservative treatments, such as physiotherapy and therapeutic ultrasound [57,58]. 

Nelson et al. and Bagnato et al., in a double-blind pilot clinical study including, respectively, 34 and 60 patients with OA treated with PEMF, showed that the VAS pain score decreased versus baseline and there was a reduction in the intake of NSAIDs [39,40]. Bagnato’s treatment scheme consisted of 12 h daily treatment for 1 month [59,60,61].

PEMF therapy is a valid option for conservatively managing several articular diseases, including early OA, patellofemoral pain syndrome, SONK and for the treatment of athletes. PEMF could be also used as a supporting therapy after an arthroscopic knee procedure or total knee arthroplasty to reduce the post-operative inflammatory state [46,47,62,63,64,65,66].

Although there is no scientific evidence about side effects and contraindications of PEMF therapy, we must underline that there are some recommended precautions in some conditions: pregnancy, since there are no clinical studies carried out on pregnant patients and pulsed electromagnetic fields emitted by the solenoids could affect the development of the foetus; pacemakers’ wearers and patients with severe arrhythmias, because PEMF could interfere with the proper functioning of the device even though there are new pacemakers on the market that have no contraindications. 

In children who have not yet completed their growth phase, PEMF should not be used because they have effects on bone calcification and increased blood circulation [64]. In other conditions such as epilepsy, acute infections, tumours, tuberculosis, mycosis, cardiopathy, wearers of magnetizable prostheses, acute viral disease, and juvenile diabetes, it is recommended to avoid applying this therapy because there is no scientific evidence to show contraindications.

## 4. Conclusions

Knee chondropathy is a very disabling pathology, especially with age. The proposal of new therapeutic approaches capable of targeting the adenosine signal, such as the intra-articular injection of PDRN and the use of Pulsed Electromagnetic Fields, has shown excellent beneficial results compared to conventional treatments and is an extra weapon in the fight against this pathology.

Biophysical stimulation has both anabolic effects on cartilage tissue and analgesic, angiogenic, and anti-inflammatory effects in the synovial microenvironment. An osteogenic effect on bone tissue and a reduction of osteoclastogenesis has been also observed. PEMFs’ short-term effects depend on their analgesic effect, the subsequent lowering of nonsteroidal anti-inflammatory drug intake, the improvement in joint range of movement, and functional time half-life. The long-term effects mainly depend on the patient’s improved quality of life. 

PEMF therapy is an emerging non-invasive approach that has a low impact on daily life routine and causes a significant increase in endogenous modulators. Experimental evidence in chondrocytes and articular cartilage suggests that adenosine replacement in the joint may be a new therapeutic approach to diseases involving cartilage damage, such as OA. The next step for future research in the clinics should focus on studying all possible uses of AR as well as limitations and side effects since there are not enough data in the literature.

Therefore, adenosine via the stimulation of the ARs and the simultaneous application of PEMF could be a valid synergic approach able to provide significant therapeutic results in different inflammatory diseases, such as the functional recovery of damaged cartilage tissues and pain control.

Although there are no studies about the beneficial and synergic effects of using intra-articular PDRN together with PEMF, their healing and regenerative capacities have been abundantly demonstrated. Furthermore, no contraindications to the synergistic use of the two treatments have been reported. Therefore, the hope for the future is that new protocols for the treatment of knee OA can be defined wherein the use of both innovative therapeutic techniques is envisaged.

## Data Availability

The data that support the findings of this study are available from the corresponding author upon reasonable request.
